# Molecular characterization of a reptarenavirus detected in a Colombian Red-Tailed Boa (*Boa constrictor imperator*)

**DOI:** 10.1186/s12985-023-02237-2

**Published:** 2023-11-15

**Authors:** Mohamed A. Abouelkhair, Ashkan Roozitalab, Ola K. Elsakhawy

**Affiliations:** Department of Biomedical and Diagnostic Sciences, University of Tennessee, Knoxville, TN USA

**Keywords:** Reptarenavirus, Colombian red-tailed Boa, *Boa constrictor imperator*, Boid inclusion body Disease, Illumina MiniSeq, L7 genotype, S6 genotype, Molecular characterization, Genetic diversity

## Abstract

The global decline in biodiversity is a matter of great concern for members of the class *Reptili*a. Reptarenaviruses infect snakes, and have been linked to various clinical conditions, such as Boid Inclusion Body Disease (BIBD) in snakes belonging to the families *Boidae* and *Pythonidae*. However, there is a scarcity of information regarding reptarenaviruses found in snakes in both the United States and globally. This study aimed to contribute to the understanding of reptarenavirus diversity by molecularly characterizing a reptarenavirus detected in a Colombian Red-Tailed Boa (*Boa constrictor imperator*). Using a metagenomics approach, we successfully identified, and *de novo* assembled the whole genomic sequences of a reptarenavirus in a Colombian Red-Tailed Boa manifesting clinically relevant symptoms consistent with BIBD. The analysis showed that the Colombian Red-Tailed Boa in this study carried the University of Giessen virus (UGV-1) S or S6 (UGV/S6) segment and L genotype 7. The prevalence of the UGV/S6 genotype, in line with prior research findings, implies that this genotype may possess specific advantageous characteristics or adaptations that give it a competitive edge over other genotypes in the host population. This research underscores the importance of monitoring and characterizing viral pathogens in captive and wild snake populations. Knowledge of such viruses is crucial for the development of effective diagnostic methods, potential intervention strategies, and the conservation of vulnerable reptilian species. Additionally, our study provides valuable insights for future studies focusing on the evolutionary history, molecular epidemiology, and biological properties of reptarenaviruses in boas and other snake species.

## Introduction

The global decline in biodiversity is a matter of great concern for members of the class *Reptilia*, comprising reptiles such as snakes, lizards, turtles, and crocodiles [1]. These diverse and ancient creatures play vital roles in ecosystems worldwide, contributing to the balance of nature and maintaining healthy habitats [2]. Boid Inclusion Body Disease (BIBD) is a potentially fatal viral disease affecting boas and pythons, characterized by the formation of eosinophilic or amphophilic intracytoplasmic inclusion bodies (IBs) within almost all cell types [3–5]. The existing body of literature indicates that several of the more than 100 known boa and python species are susceptible to BIBD [5]. BIBD can lead to progressive and degenerative clinical signs, including neurologic abnormalities such as star-gazing and head tremors, as well as respiratory signs like mouth breathing and increased respiratory effort [5, 6]. Unfortunately, there is currently no known curative therapy for BIBD, emphasizing the need for a deeper understanding of the underlying viral agents and their characteristics [7]. Previous studies have reported the association between reptarenaviruses and Boid Inclusion Body Disease in various snake species, highlighting the importance of further investigations [6, 8].

The family *Arenaviridae* currently comprises five genera, namely, *Antennavirus, Hartmanivirus, Innmovirus, Mammarenavirus,* and *Reptarenavirus* [9]. Viruses in the genus *Reptarenavirus* infect snakes, and some reptarenaviruses cause boid inclusion body disease (BIBD) [8–10].

The genome of reptarenaviruses produce enveloped virions containing bisegmented negative-sense RNA with an ambisense coding strategy [9]. The long (L) segment encodes the zinc finger matrix protein (ZP) and RNA-directed RNA polymerase (RdRp) [9]. The short (S) segment of the genome codes for the glycoprotein precursor (GPC) and the nucleoprotein (NP) [9]. For segmented RNA viruses like arenaviruses, the potential for generating novel genotypes is substantial due to their unique genetic makeup. These viruses can undergo mutation, recombination, and reassortment, processes that contribute to their genetic diversity and may ultimately lead to the emergence of a new lineage of arenaviruses [11]. Moreover, the occurrence of recombination and reassortment events in natural arenavirus infections has been definitively documented through genetic analyses. Such genetic exchanges can lead to the emergence of novel strains with altered pathogenic properties or expanded host ranges, underscoring the importance of monitoring and understanding these processes for effective surveillance and control of arenavirus infections [10].

Historically, the detection and identification of reptarenaviruses have been challenging due to the lack of specific diagnostic assays and the limited availability of complete viral genome sequences. However, recent advances in metagenomics approaches have revolutionized the field of viral discovery and characterization [12]. Metagenomics techniques allow for the identification and sequencing of viral genomes directly from clinical samples, bypassing the need for virus isolation or culture. These methods involve the extraction of total nucleic acids from the sample, followed by next-generation sequencing and bioinformatics analysis to identify and assemble viral sequences [13, 14]. Such approaches have proven to be particularly effective in uncovering novel viral agents, including reptarenaviruses, and elucidating their genetic composition [7, 15, 16].

The objective of this study was to employ a metagenomics approach to identify and molecularly characterize a reptarenavirus in a Colombian Red-Tailed Boa with BIBD symptoms. Such information is vital for development of diagnostic assays for early detection and monitoring of viral infections in snake populations. Furthermore, it provides valuable insights for the implementation of effective preventive strategies to mitigate the transmission and spread of Boid Inclusion Body Disease among snakes.

## Materials and methods

### Sample collection

Blood sample was collected from the tail vein of a 2-year-old female Colombian Red-Tailed Boa that was presented to the Avian and Exotics department at the University of Tennessee College of Veterinary Medicine (UTCVM). The snake was brought in due to clinical signs of whole-body twitching and excessive yawning.

### Reverse transcription-polymerase chain reaction (RT-PCR) screening

The RNA was extract from the blood sample and purified using MagMAX™ Viral/Pathogen Nucleic Acid Isolation Kit (Thermo Fisher Scientific, USA). The RNA was reverse transcribed into cDNA using SuperScript™ III Reverse Transcriptase (Thermo Fisher Scientific, USA) and random hexamer oligonucleotide [10]. Diluted cDNA was used as template in RT-PCR reactions using degenerate primers targeting the glycoprotein gene, as previously reported by other researchers [10].

### Library preparation and sequencing

The nucleic acid was extracted and purified using MagMAX™ Viral/Pathogen Nucleic Acid Isolation Kit (Thermo Fisher Scientific, USA). Quantity and quality of the RNA were assessed using a NanoDrop 2000 spectrophotometer (Thermo Fisher Scientific, USA) and Qubit fluorometer (Fisher, Waltham, MA). Host DNA was removed by DNase treatment using heat-labile Double-Strand Specific DNase (ArcticZymes) followed by inactivation at 58 °C for 5 min.

For the first strand cDNA synthesis, the ProtoScript® II First Strand cDNA Synthesis Kit (New England Biolabs, MA, USA) was used following the manufacturer’s instructions. For the second strand cDNA synthesis, the NEBNext® Ultra™ II Directional RNA Second Strand Synthesis Module (New England Biolabs, MA, USA) was used following the manufacturer’s instructions. Sequencing libraries were prepared using the Nextera DNA Flex kit (Illumina, Inc., USA) according to the manufacturer’s instructions. Paired-end 2 × 150 bp sequencing was performed on an Illumina Miniseq in the virology and molecular diagnostic laboratory at the University of Tennessee, Knoxville TN, USA.

### Sequence analysis

The sequence analysis was performed using the Sunbeam pipeline (version 3.1.1), a comprehensive and flexible bioinformatics tool for metagenomic data analysis [17]. First, the raw sequence reads obtained from the metagenomic sequencing of the reptarenavirus-infected Colombian Red-Tailed Boa were subjected to quality control using FastQC (version 0.12.1) [18] to assess read quality and detect any potential issues or biases. Adapter trimming and quality filtering were performed using Cutadapt (version 4.4) [19] and Trimmomatic (version 0.39) [20] to remove low-quality bases and adapter sequences. Sequence complexity in each read was assessed using Komplexity (version 0.3.6). Following the quality control steps, the preprocessed reads were classified taxonomically using Kraken 2 (version 2.1.3) [21]. Taxonomic classification was performed using Kraken 2 on preprocessed Illumina reads. Kraken 2 represents the most recent iteration of the Kraken taxonomic classification system. It employs precise *k-mer* matching to achieve rapid and accurate classification. In this process, Kraken constructs an index of all *k-mers* found in the reference genomes and assigns each *k-mer* to the least common ancestor (LCA) of all species possessing that particular *k-mer*. Then Kraken matches the *k-mers* contained in the reads to this index and eventually assigns the reads to the taxon with the most fitting *k-mers* by following the path from the root of the tree [21]. To construct a standard database, which includes RefSeq complete bacterial, archaeal, and viral genomes, the human genome, and a collection of known vectors (UniVec_Core), the process utilized 32 threads with default parameters. These operations were conducted on a Jetstream2 instance, specifically an m3.xl storage optimized instance with 125 gigabytes (GB) of RAM [22]. The Kraken2 result was subsequently visualized using Pavian with rank codes representing the taxonomic ranks of domain (D), kingdom (K), phylum (P), family (F), or genus (G) [23].

Then viral reads were assembled into contigs using MEGAHIT (version 1.2.9) [24]. All of the sequence analysis described in this study was conducted utilizing the Jetstream2 cloud computing resource which is supported by the National Science Foundation [22]. The assembled contigs and sequences were edited and aligned with Geneious Prime® v.2023.1.2 [25]. The open reading frame (ORF) of the viral genome was predicted using ORF finder at NCBI (https://www.ncbi.nlm.nih.gov/orffinder, accessed 15 June 2023) with default parameters. We used the Pairwise Sequence Comparison (PASC) web tool [26] (https://www.ncbi.nlm.nih.gov/sutils/pasc/viridty.cgi?textpage=overview, accessed 20 July 2023), recommended by the *Arenaviridae* study group of the International Committee on Taxonomy of Viruses (ICTV) for arenavirus classification [26–28], to analyze the identified reptarenavirus segments.

### Phylogenetic analysis

L segment nucleic-acid sequences corresponding to the reptarenavirus genotypes L1 to L23 (Table 1), as well as nucleic-acid sequences corresponding to reptarenavirus genotypes S1 to S11 were retrieved from GenBank (Table 2). These sequences were then aligned with the nucleotide sequences of the reptarenavirus identified in our study using the MAFFT E-INS-i algorithm. Then, phylogenetic trees were inferred for each segment using the maximum-likelihood (ML) method implemented in MEGA11 (version 11.0.13) under the GTR + I + Γ4 nucleotide substitution model as determined by the best model finder in MEGA 11 [29]. The node supports were estimated using 1000 bootstrap replicates.

**Table 1:** Reptarenavirus L segment genotypes and their corresponding accessions numbers retrieved from GenBank database

Genotype	GenBank Accession	strain
L1	KP071533.1	Reptarenavirus/*Corallus annulatus*/California/snake1/2009
L2	KP071525.1	Reptarenavirus/*Boa constrictor*/California/snake5/2009
L3	KP071569.1	Reptarenavirus/*Boa constrictor*/Florida/snake34/1997
L4	KP071546.1	Reptarenavirus/*Boa constrictor*/California/snake33/2008
L5	KP071489.1	Reptarenavirus/*Boa constrictor*/California/snake47/2013
L6	KP071656.1	Reptarenavirus/*Boa constrictor*/Georgia/snake29/2007
L7	KP071566.1	Reptarenavirus/*Boa constrictor*/Florida/snake34/1997
L8	KP071511.1	Reptarenavirus/*Boa constrictor*/California/snake35/2013
L9	KP071563.1	Reptarenavirus/*Boa constrictor*/Florida/snake34/1997
L10	KP071517.1	Reptarenavirus/*Boa constrictor*/Georgia/snake25/2005
L11	KP071623.1	Reptarenavirus/*Boa constrictor*/California/snake40/2010
L12	KP071550.1	Reptarenavirus/*Boa constrictor*/California/snake33/2008
L13	KP071574.1	Reptarenavirus/*Boa constrictor*/Arkansas/snake37/2012
L14	KP071562.1	Reptarenavirus/*Boa constrictor*/Florida/snake34/1997
L15	KP071676.1	Reptarenavirus/*Boa constrictor*/Florida/snake32/2005
L16	KP071614.1	Reptarenavirus/*Boa constrictor*/Louisiana/snake41/2010
L17	KP071560.1	Reptarenavirus/*Boa constrictor*/Florida/snake34/1997
L18	KP071479.1	Reptarenavirus/*Boa dumerili*/California/snake27/2009
L19	KP071548.1	Reptarenavirus/*Boa constricto*r/California/snake33/2008
L20	KP071622.1	Reptarenavirus/*Boa constrictor*/California/snake40/2010
L21	KP071567.1	Reptarenavirus/*Boa constrictor*/Florida/snake34/1997
L22	KP071561.1	Reptarenavirus/*Boa constri*ctor/Florida/snake34/1997
L23	KP071674.1	Reptarenavirus/*Boa constrictor*/Florida/snake32/2005

**Table 2:** Reptarenavirus S segment genotypes and their corresponding accessions numbers retrieved from GenBank database

Name	GenBank Accession	strain
S1	KP071532.1	Reptarenavirus/*Corallus annulatus*/California/snake1/2009
S2	KP071541.1	Reptarenavirus/*Boa constrictor*/Tennessee/snake6/2008
S3	KP071630.1	Reptarenavirus/*Boa constrictor*/California/snake7/2008
S4	KP071474.1	Reptarenavirus/*Boa dumerili*/California/snake27/2009
S5	KP071583.1	Reptarenavirus/*Boa constrictor*/Louisiana/snake44/2010
S6	KP071573.1	Reptarenavirus/*Boa constrictor*/Arkansas/snake37/2012
S7	KP071578.1	Reptarenavirus/*Boa constrictor*/Florida/snake26/2011
S8	KP071509.1	Reptarenavirus/*Boa constrictor*/California/snake35/2013
S9	KP071671.1	Reptarenavirus/*Boa constrictor*/Georgia/snake19/2007
S10	KP071473.1	Reptarenavirus/*Boa dumerili*/California/snake27/2009
S11	KP071559.1	Reptarenavirus/*Boa constrictor*/Florida/snake34/1997
UGV-1	NC_039005.1	University of Giessen virus 1 (UGV-1)

The amino acid sequences of the RdRp proteins associated with reptarenavirus genotypes L1 to L23, as well as the amino acid sequences of the NP and GPC proteins associated with reptarenavirus genotypes S1 to S11, were retrieved from GenBank. These sequences were then aligned with the amino acid sequences of the reptarenavirus identified in our study using the MAFFT E-INS-i algorithm. Maximum likelihood phylogenies were created using PhyML plugin for Geneious Prime® (version 2023.2.1), 100 bootstrap replicates, and otherwise default parameters.

## Results

### Detection of reptarenavirus RNA

In boas, antemortem BIBD diagnosis relies on the detection of inclusion bodies (IBs) in histological specimens or blood smears [5, 15, 30–35]. Nonetheless, studies have demonstrated that not all cases of reptarenavirus infection result in BIBD, with some infected snakes remaining free of intracytoplasmic inclusion bodies (IBs) and clinically healthy for an extended period, often spanning years [4, 30, 31, 33, 36–38]. In such cases, molecular-based diagnostic methods could offer an alternative, providing sensitive and specific tests for accurate detection [39]. As a result, we only performed reverse transcription-PCR (RT-PCR), which yielded an amplicon of the correct size, consistent with the target gene.

### Taxonomic classification results using Kraken 2

**Fig. 1:**
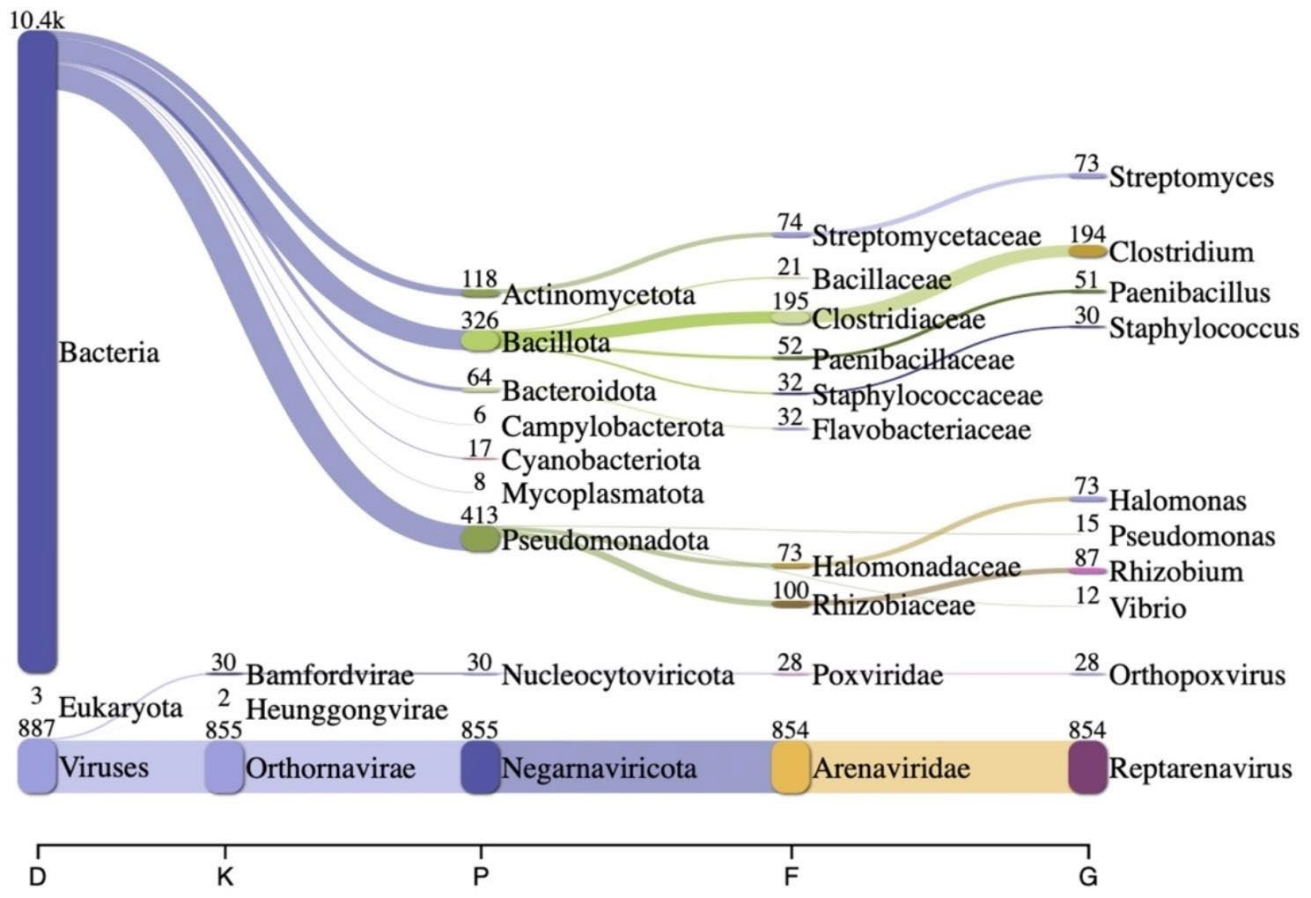
Sankey diagrams of Kraken 2 report. The width of the flow is proportional to the number of reads. The number above each node is the number of *k-mer* hits. A rank code, indicating domain (D), phylum (P), family (F), or genus (G) was used. Out of the total 887 viral reads analyzed, a substantial proportion of 854 reads (96.3%) were classified as reptarenavirus

Metagenomic sequencing was conducted on the clinical samples obtained from the Colombian Red-Tailed Boa. Out of the total 887 viral reads analyzed, a substantial proportion of 854 reads (96.3%) were classified as reptarenavirus Fig. 1.

### Open reading frame analysis and genome organization

The sequence reads obtained from our dataset were assembled into two distinct segments: a large (L) segment (7019 base pairs (bps)) and a small (S) segment (3352 bps). The complete genome sequence for L and S segments was deposited in NCBI (https://www.ncbi.nlm.nih.gov/) under accession numbers OR194165 and OR194166, respectively. Stenglein et al. [15] reported that the L and S segments of reptarenaviruses have the capacity to recombine and reassort within their host, leading to the emergence of different genotypes. Specifically, they identified S1-11 and L1-23 segment genotypes in the reptarenaviruses sequenced in the USA, indicating the genetic plasticity and evolution of these viruses within their host populations. Within genotypes, the L segment sequences showed a mean pairwise nucleotide identity of 96%, while between genotypes, the sequences shared 65% identity. For the S segments, the mean pairwise nucleotide identity within genotypes was 96%, and between genotypes, it was 64%.

We used the Basic Local Alignment Search Tool (BLAST) (https://blast.ncbi.nlm.nih.gov/Blast.cgi, accessed 15 April 2023) and the Pairwise Sequence Comparison (PASC) web tool [26] (https://www.ncbi.nlm.nih.gov/sutils/pasc/viridty.cgi?textpage=overview, accessed 20 July 2023), recommended by the *Arenaviridae* study group of the International Committee on Taxonomy of Viruses (ICTV) for arenavirus classification [26–28], to analyze the identified reptarenavirus segments.

The analysis showed that the Colombian Red-Tailed Boa in this study carried the University of Giessen virus (UGV-1) S or S6 (UGV/S6) segment (BLAST, 97.88% identical to NC_039005.1 and 97.02% identical to KP071573.1) and L genotype 7 (PASC, 97.2% identical to KP071566.1; BLAST, 98.77% identical to KP071566.1). The PASC tool for genotyping the S segment was not operational at the time of writing this manuscript.

### Phylogenetic analysis

**Fig. 2:**
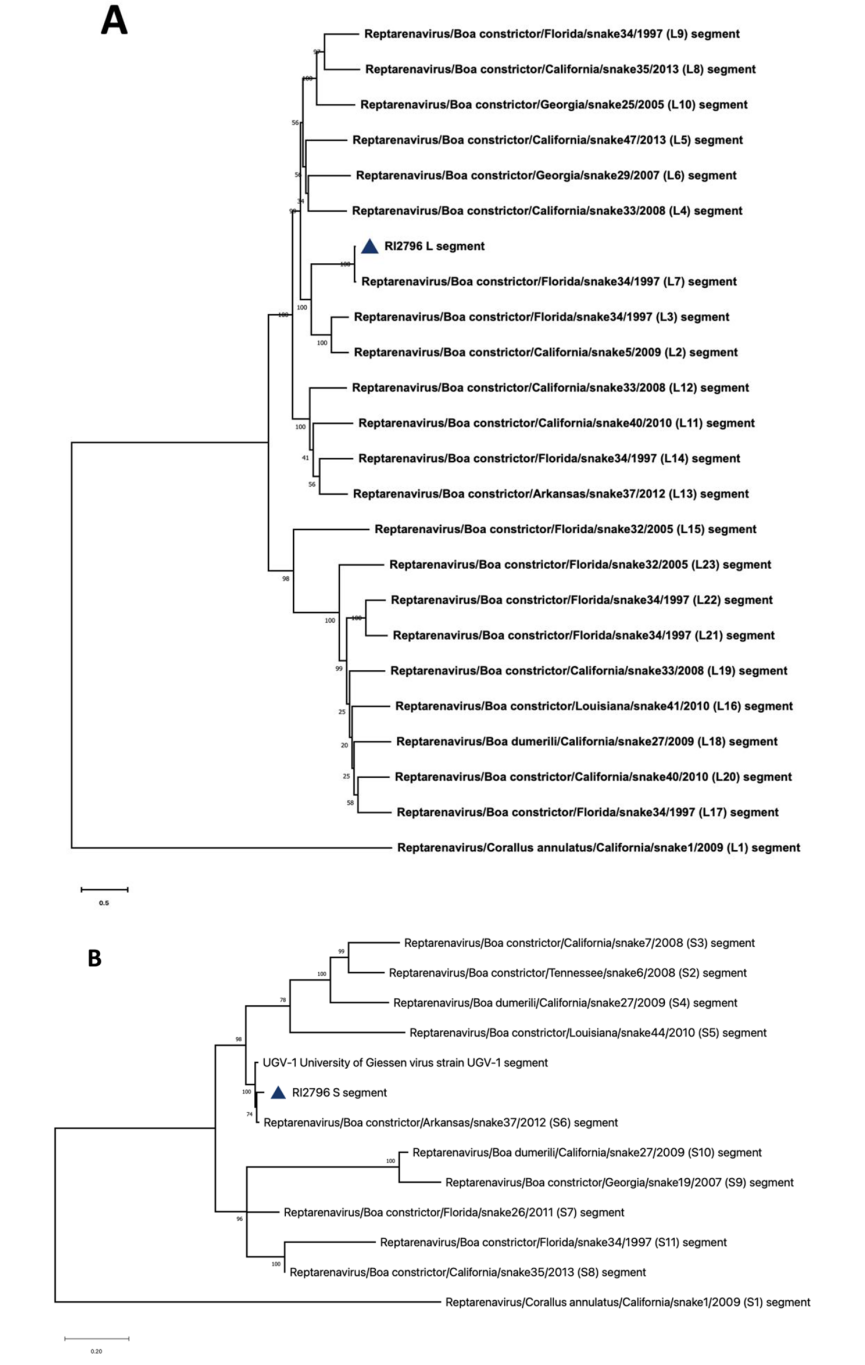
(**A**) Phylogenetic tree based on the reptarenavirus complete L segments, with ML and Bayesian methods, using the evolutionary model GTR + G + l. (**B**) Phylogenetic tree based on the reptarenavirus complete S segments, with ML and Bayesian methods, using the evolutionary model GTR + G + l. Evolutionary analysis was conducted in MEGA 11. Blue triangles indicate the sequences identified in this study. Numbers above branches indicate bootstrap values

**Fig. 3:**
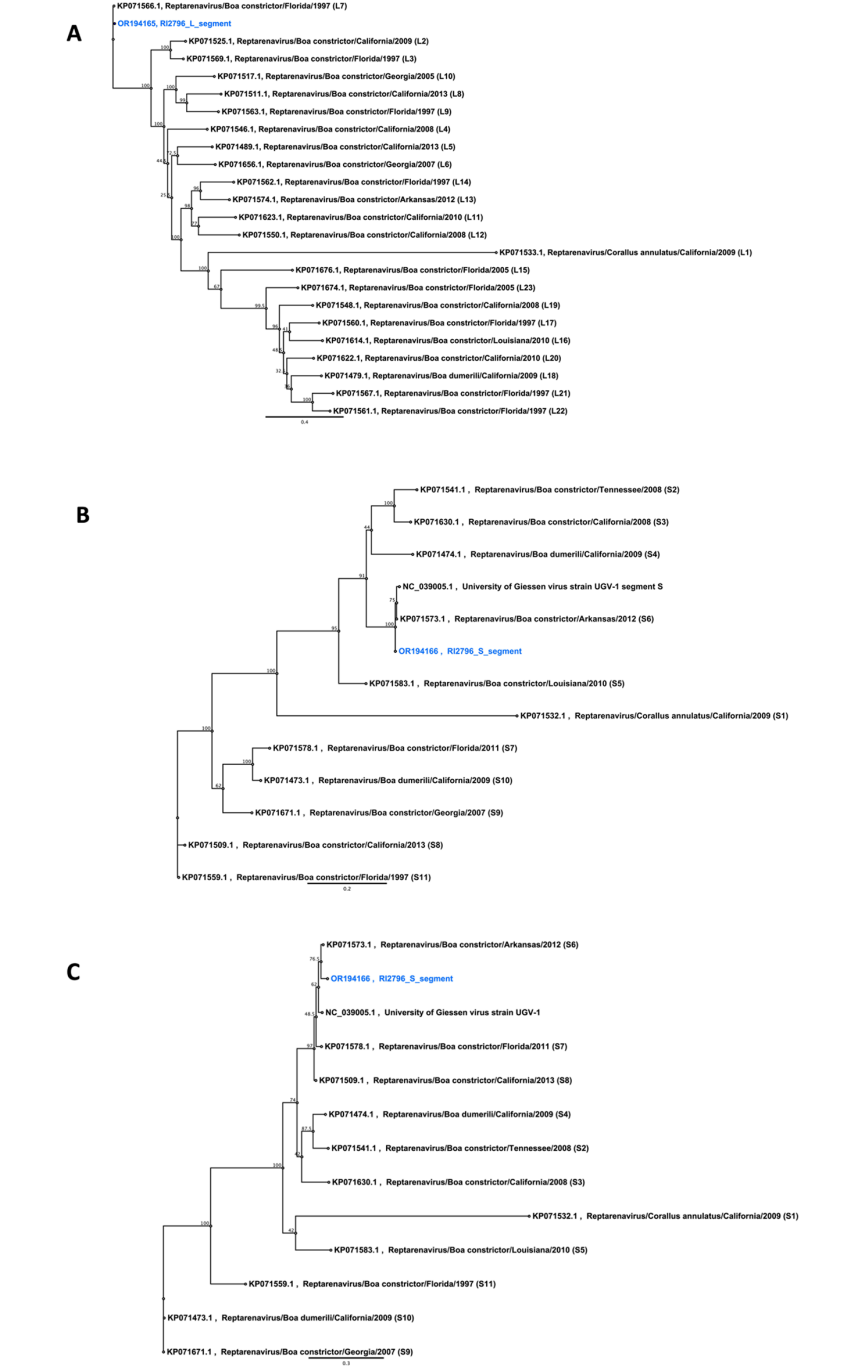
Phylogenetic analysis of Reptarenavirus RdRps, NPs, and GPCs. (**A**) A phylogenetic tree based on the RdRp amino acid sequences of the viruses identified in this study and those available in GenBank. (**B**) Phylogenetic tree based on the NP amino acid sequences of the viruses identified in this study and those available in GenBank. (**C**) A phylogenetic tree based on the GPC amino acid sequences of the viruses identified in this study and those available in GenBank. RdRp, NP, and GPC amino acid sequences from this study are highlighted in blue. Numbers above branches indicate bootstrap values

The phylogenetic analysis revealed that RI2796 L segment clustered tightly with the L7 genotype sequences (Fig. 2A) while RI2796 S segment clustered tightly with the University of Giessen virus (UGV-1) or S6 (UGV/S6) genotype (Fig. 2B). The clustering of RI2796 S segment with the UGV/S6 genotype in our analysis is consistent with these earlier findings, further reinforcing the prevalence and persistence of the UGV/S6 genotype within reptarenavirus populations. Phylogenetic analysis of Reptarenavirus RdRps (Fig. 3A), NPs (Fig. 3B), and GPCs (Fig. 3C) confirm that the Colombian Red-Tailed Boa of this study appeared to carry only a single pair of L and S segments.

## Discussion

The first comprehensive conservation assessment of reptiles found that 21.1% of the animals were classed as Vulnerable, Endangered or Critically Endangered [40]. This is significantly more than birds, of which 13.6% are threatened [40]. Reptarenaviruses cause Boid Inclusion Body Disease (BIBD), a potentially fatal disease occurring in boas and pythons across the globe. This makes the study of reptarenaviruses all the more crucial, as BIBD poses a significant threat to these reptile species and the ecosystems they inhabit [38]. However, there is a lack of comprehensive information regarding reptarenaviruses isolated from snakes. Thus, this study aimed to contribute to the understanding of reptarenavirus diversity by applying a metagenomic approach to characterize a reptarenavirus in a Colombian Red-Tailed Boa (*Boa constrictor imperator*) with BIBD symptoms. Furthermore, this study underscores the importance of employing metagenomic sequencing as a diagnostic tool to detect highly divergent viruses, particularly within the *Arenaviridae* family.

In boas, antemortem BIBD diagnosis relies on the detection of inclusion bodies (IBs) in histological specimens or blood smears [5, 15, 30–35]. Nonetheless, studies have demonstrated that not all cases of reptarenavirus infection result in BIBD, with some infected snakes remaining free of intracytoplasmic inclusion bodies (IBs) and clinically healthy for an extended period, often spanning years [4, 30, 31, 33, 36–38]. To address this complexity, Thiele, Tanja et al. proposed a classification system comprising three categories: reptarenavirus infection (IB-negative), nonclinical BIBD (IB-positive without clinical disease), and clinical BIBD (IB-positive with clinical disease) [39]. Snakes with nonclinical BIBD and those serving as asymptomatic carriers of reptarenaviruses act as a hidden reservoir of infection for other animals, facilitating the introduction, spread, and persistence of these viruses within colonies. This emphasizes the critical need for the development of a precise and sensitive molecular diagnostic method to screen these clinically healthy carriers for the presence of reptarenaviruses [39].

By utilizing a metagenomics approach, we successfully identified and assembled the complete genomic sequences of a reptarenavirus isolated from a Colombian Red-Tailed Boa. Metatranscriptomic analysis revealed the presence of one reptarenavirus L segment (L7 genotype) and one reptarenavirus S segment (UGV/S6 genotype). This observation is consistent with previous studies that have consistently reported the prevalence and persistence of the UGV/S6 genotype across different reptilian populations. The dominance of the UGV/S6 genotype suggests that it possesses certain advantageous traits or adaptations, enabling it to outcompete other genotypes within the host population. Several factors may contribute to the observed dominance of the UGV/S6 genotype. Firstly, genetic factors such as mutations or genetic variations may confer a selective advantage to the UGV/S6 genotype, enabling it to replicate more efficiently or evade the host immune response effectively. Additionally, viral factors, including virulence factors or replication strategies, might play a role in the competitive advantage of the UGV/S6 genotype. Future research is needed to investigate the molecular mechanisms underlying this dominance and identify the specific genetic or viral factors involved.

The Colombian Red-Tailed Boa of this study appeared to carry only a single pair of L and S segments. The current knowledge on reptarenaviruses presents a very different scenario in which the majority of snakes with BIBD carry several L and S segments of different genotypes [7, 8, 10, 32, 33]. The identification of multiple genotypes within the same snake provides evidence for the potential coinfection and coexistence of reptarenaviruses. These coinfections may result from encounters with multiple viral strains or subsequent infections occurring in snakes already carrying one genotype or it may be due to a sampling bias [39]. The coexistence of multiple reptarenavirus subtypes in snakes highlights the complex dynamics and potential for viral interactions within reptile host populations [32, 37].

This observation also raises questions regarding the consequences of reptarenavirus coinfections in snakes. Coinfections may influence viral pathogenesis, host immune responses, and viral evolution. Further investigations are warranted to elucidate the impact of these coinfections on the health and fitness of snakes, as well as their implications for the transmission and spread of reptarenaviruses within snake populations.

## Conclusion

This study represents a significant contribution to the field by identifying and characterizing a reptarenavirus in a Colombian Red-Tailed Boa. The detection of this novel virus not only enhances our knowledge of viral diversity in reptilian species but also sheds light on the evolutionary relationships within the *Reptarenavirus* genus. Further investigations are warranted to determine the prevalence, pathogenicity, and zoonotic potential of these viruses. Understanding their impact on both captive and wild reptile populations is of utmost importance. Moreover, future research should focus on developing diagnostic assays for early detection, implementing preventive strategies, and elucidating the mechanisms of transmission. By unraveling the mysteries surrounding these reptarenaviruses, we can better safeguard the health and conservation of reptiles, as well as mitigate potential risks to human health.

## Data Availability

The complete genome sequence for L and S segments was deposited in NCBI (https://www.ncbi.nlm.nih.gov/) under accession numbers OR194165 and OR194166, respectively (BioProject accession number PRJNA982190). The corresponding raw reads from the Illumina sequencing are available in the Sequence Read Archive (SRA) under the accession number SRR24908467.
